# A dual function fusion protein of Herpes simplex virus type 1 thymidine kinase and firefly luciferase for noninvasive *in vivo *imaging of gene therapy in malignant glioma

**DOI:** 10.1186/1479-0556-2-7

**Published:** 2004-08-04

**Authors:** Ariane Söling, Christian Theiß, Stephanie Jungmichel, Nikolai G Rainov

**Affiliations:** 1 Molecular Neurooncology Laboratory, Dept. Neurosurgery, Martin-Luther-University Halle-Wittenberg, 06097 Halle, Germany; 2 Dept. Neurological Science, University of Liverpool, Liverpool 9L 7LJ, United Kingdom

**Keywords:** glioma, bioluminescence imaging, gene therapy, herpes simplex virus type 1 thymidine kinase, luciferase

## Abstract

**Background:**

Suicide gene therapy employing the prodrug activating system Herpes simplex virus type 1 thymidine kinase (HSV-TK)/ ganciclovir (GCV) has proven to be effective in killing experimental brain tumors. In contrast, glioma patients treated with HSV-TK/ GCV did not show significant treatment benefit, most likely due to insufficient transgene delivery to tumor cells. Therefore, this study aimed at developing a strategy for real-time noninvasive *in vivo *monitoring of the activity of a therapeutic gene in brain tumor cells.

**Methods:**

The *HSV-TK *gene was fused to the firefly luciferase (*Luc*) gene and the fusion construct *HSV-TK-Luc *was expressed in U87MG human malignant glioma cells. Nude mice with subcutaneous gliomas stably expressing HSV-TK-Luc were subjected to GCV treatment and tumor response to therapy was monitored *in vivo *by serial bioluminescence imaging. Bioluminescent signals over time were compared with tumor volumes determined by caliper.

**Results:**

Transient and stable expression of the HSV-TK-Luc fusion protein in U87MG glioma cells demonstrated close correlation of both enzyme activities. Serial optical imaging of tumor bearing mice detected in all cases GCV induced death of tumor cells expressing the fusion protein and proved that bioluminescence can be reliably used for repetitive and noninvasive quantification of HSV-TK/ GCV mediated cell kill *in vivo*.

**Conclusion:**

This approach may represent a valuable tool for the *in vivo *evaluation of gene therapy strategies for treatment of malignant disease.

## Background

Treatment with the suicide gene/ prodrug activating system herpes simplex virus type I thymidine kinase/ ganciclovir (HSV-TK/ GCV) is highly efficient in animal models of malignant glioma [1-3]. In contrast, clinical trials employing the HSV-TK/ GCV system and a retroviral vector have demonstrated only a limited effect in glioblastoma patients [4-8], implying that transfer and distribution of the transgene in human brain tumors were very low *in vivo *and differed obviously from the findings in animal experiments. Presently, the standard method for assessing delivery of therapeutic transgenes to tumors relies on *ex vivo *analysis of explanted tumor tissue [7,9,10]. Time course analysis of transgene expression thus requires a multitude of animals to be sacrificed. In the past few years, noninvasive imaging techniques such as positron emission tomography (PET), magnetic resonance imaging, and optical imaging methods using fluorescence and bioluminescence were introduced and increasingly used for temporal and spatial monitoring of transgene expression [11-13].

Bioluminescence imaging (BLI) using luciferase (*Luc*) from the North American firefly *Photinus pyralis *as a reporter has several advantages compared to other imaging methods: (1) the technique is very sensitive (possibly 10^-15 ^– 10^-17 ^mole of luciferase/L are detectable *in vivo*, [13]) and detects tumor cells at a stage where radiography and PET cannot [14,15], (2) bioluminescence imaging using a cooled CCD camera does not require great technical expertise and (3) it is faster and less expensive than many other imaging techniques. Furthermore, in contrast to fluorescence imaging, where autofluorescence may interfere with the signal of interest [13], background luminescence is negligible.

This study aimed at generating a sensitive tool for noninvasive *in vivo *monitoring of the activity of a therapeutic transgene by fusing the bioluminescent reporter gene *Luc *to the bioactivating "suicide" gene *HSV-TK*. We investigated whether this fusion construct could be used to monitor HSV-TK mediated cytotoxicity in malignant glioma by serial optical imaging *in vivo*. Noninvasive real time evaluation of localization, activity and persistence of a therapeutic gene in living animals may represent an important step towards optimization of gene therapy protocols.

## Methods

### Vector construction

The *HSV-TK *cDNA from the retroviral vector G1Tk1SvNa ([16], kind gift from E. Otto, GTI Inc., Gaithersburg, MD) and the "humanized" firefly luciferase (*Luc*) gene from the pGL3 vector (Promega) were ligated into pCDNA 3.1(-) (Invitrogen). For the fusion construct, EGFP in the pEGFPLuc vector (BD Biosciences) was exchanged for *HSV-TK *cDNA, which had been amplified from G1Tk1SvNa by PCR. The resulting *HSV-TK-Luc *fusion gene contained a humanized form of the firefly *Luc *gene to ensure high expression in mammalian cells [17]. The full length *HSV-TK *cDNA was inserted in frame upstream of the *Luc *cDNA, and both genes were separated by a linker sequence of 33 nucleotides. All transgenes were expressed under the control of the CMV promoter. The correct sequence of the fusion construct *HSV-TK-Luc *was confirmed by DNA sequencing.

### Cell culture and transfection

The human glioblastoma cell lines U87MG, T98G, LN18, U343, LN-Z308 and human embryonic kidney 293 cells were cultured under standard conditions. Cells were seeded in 6-well plates at a density of 3 – 5 × 10^5 ^cells/ well 16 to 24 h prior to transfection. Cells were transfected under serum-free conditions with the indicated amounts of DNA and Lipofectamine (Invitrogen) according to the manufacturer's protocol. For selection of stable clones transfected cells were replated at low density 48 h after transfection and incubated with 1 mg/ml (final concentration) geneticin (Calbiochem, Bad Soden, Germany) for 4 weeks. Colonies were picked and analyzed for transgene expression.

### Cytotoxicity assay

Transiently or stably transfected U87MG cells were seeded at 4 × 10^3 ^cells/ well in a 96-well plate. GCV was added at final concentrations of 0 – 10 μg/ml and cells were incubated at 37°C/ 5% CO_2 _for 4 days. MTT (Sigma, Deisenhofen, Germany) was added at a final concentration of 0.5 mg/ml for 2 h. Absorbance was measured in a microplate reader (Victor2, Perkin Elmer Life Sciences, Turku, Finland) at 590 nm (reference 660 nm). Experiments were performed in quadruplicates and repeated at least twice. Results are reported along with the standard deviation (SD).

### Cell culture assays for luciferase activity

Transiently transfected U87MG cells were lysed in CCLR lysis buffer (Promega) 2 days after transfection. Stably transfected cells were lysed in the same buffer when they had reached ~90% confluence. Protein content of all cell lysates was determined by the Bradford Protein assay (Bio-Rad, Munich, Germany). Equal amounts of protein were analyzed luminometrically for luciferase activity with a microplate reader (Victor2) using the Luciferase Assay System reagent (Promega). All experiments were repeated at least twice and mean values are reported along with the SD.

For bioluminescence imaging of intact cells HSV-TK-Luc expressing U87 glioma cells were transferred to a black microtiter plate in order to minimize light scattering, and MTT assay was performed in quadruplicates as described above. On day 4 after addition of GCV, D-Luciferin was added to a final concentration of 500 μM to the culture medium. Cells were placed in a dark box and light emission was imaged using a cooled CCD camera (Visiluxx Imager, Visitron). Light emitted from a region of interest (ROI) drawn over each well was quantified and mean values from quadruplicate measurements were compared with MTT results.

### Immunohistochemistry and Hematoxylin-Eosin (HE) staining

Immunohistochemistry on paraffin sections using a rabbit polyclonal anti-Luc antibody (CR2029RAP, Europa Bioproducts) was performed essentially as described by Lee et al [18]. HE staining was performed according to standard protocols.

### Animal experiments

All animal protocols were approved by the Animal Care and Use Committee at Martin-Luther-University Halle-Wittenberg. Six week old male NMRI nu/nu mice (Charles River) were injected s.c. at four sites, each with 2 × 10^6 ^human U87MG glioma cells stably expressing the HSV-TK-Luc fusion protein. When xenografts had reached a size of ~5 mm in diameter, in general on days 7 to 9 post tumor implantation GCV therapy was initiated. Mice were injected twice daily i.p. with 30 mg/ kg GCV for 14 days. Control mice with xenografts (n = 3) received saline injections. Tumor size was measured every 2 to 4 days by caliper. Tumor volume was calculated according to the formula 0.52 × width^2 ^× length.

### Bioluminescence imaging

For BLI animals were anesthetized with ketamine/xylazine and injected i.p. with 150 mg/ kg D-Luciferin. Approximately 8 minutes after D-Luciferin injection mice were placed in a dark box and a grayscale image was acquired at low light (exposure time 2 seconds). Bioluminescence was measured in the dark by a CCD camera cooled to -120°C (VisiLuxx Imager), using an acquisition time of 15 min and binning 6. Bioluminescent signals were displayed in pseudocolors and superimposed on the grayscale image using Metamorph software (Visitron). Mice receiving GCV were imaged at least on days 7, 15, 22, 29, and 56 post tumor implantation (corresponding to start and day 8 of GCV therapy, as well as days 1, 8, and 35 after end of GCV therapy), while untreated control animals were subjected to BLI on days 7, 22, 29 and 35. In each animal a region of interest (ROI) was drawn over a single tumor or over all tumors as indicated in the text. Integrated as well as maximum light units (= counts) within this area were calculated after background subtraction. Final values are reported as the mean of the integrated or maximum counts obtained from all mice within one group. The CCD camera in use has a quantum efficiency approaching 90% at wavelengths between 550 and 770 nm, indicating that one photon is converted to ~0.9 electrons. One photoelectron corresponds to 4.52 counts. For serial quantification of light emission the conditions for image acquisition (e.g. exposure time, time between D-Luciferin application and image acquisition, stage position) were kept constant.

### Statistics

Statistical analysis was performed using the ANOVA and Student's t test (SPSS and Microcal Origin Software). A p value of <0.05 was considered significant.

## Results

### Characterization of the HSV-TK-Luc fusion construct

To achieve a strictly equimolar coexpression of a therapeutic and a reporter gene, the *HSV-TK *cDNA was fused in frame with the *Luc *cDNA in 2 ways: one fusion protein contained HSV-TK N-terminally, in the other construct Luc preceded the HSV-TK moiety. Both constructs were expressed under control of the CMV promoter. Several human glioma cell lines as well as 293 cells were transiently transfected with these constructs. In general, Luc activity was found to be up to 50-fold higher in cells expressing the HSV-TK-Luc construct compared to cells expressing the Luc-HSV-TK construct (data not shown). Therefore, all further studies were performed with the HSV-TK-Luc fusion construct.

**Figure 1:**
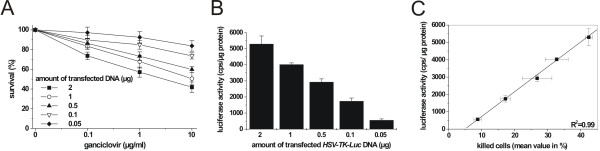
Cytotoxic and bioluminescent activity in U87MG glioma cells transiently transfected with different amounts of *HSV-TK-Luc *plasmid. (A) Cytotoxic activity as measured by MTT assay and (B) luciferase activity as determined luminometrically in cell lysates. Results are displayed as counts per second (cps)/ μg protein. (C) Linear regression analysis of cytotoxic activity plotted against luciferase activity for the different amounts of plasmid DNA. Results from 3 independent experiments were used.

**Figure 2:**
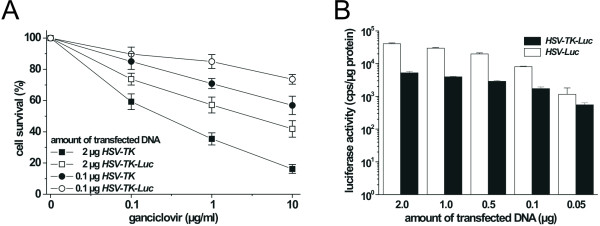
Comparison of the enzymatic activities of the HSV-TK-Luc fusion protein, HSV-TK, and Luc. U87MG cells were transiently transfected with 0.05 – 2 μg of *HSV-TK-Luc*, *Luc*, or *HSV-TK *plasmid. Cells transfected with equimolar amounts of DNA were analyzed for cytotoxic and bioluminescent activity. (A) Cytotoxic activity of cells transfected with *HSV-TK-Luc *or *HSV-TK*. For reasons of clarity only the graphs for 0.1 μg and 2 μg of transfected DNA are shown. (B) Luciferase activity in U87MG cells transfected with *HSV-TK-Luc *or *Luc *only. Results represent 3 independent experiments.

In order to characterize this fusion construct more thoroughly, both transient and stable transfection experiments were performed using the human U87MG glioma cell line (Figures 1 and 2). For transient transfection experiments, cells were transfected with 50 ng (~11.2 fmol) – 2 μg (~450 fmol) of plasmid DNA harboring either *HSV-TK*, *Luc*, or *HSV-TK-Luc *transgenes, respectively. As all 3 vectors were of equal size, equal amounts of DNA corresponded to equimolar amounts of plasmid. Cytotoxic activity as measured by MTT assay was compared to luminometrically determined light production and found to be tightly correlated in *HSV-TK-Luc *transfected cells (Figure 1, R^2 ^= 0.99; p < 0.0001). Photon emission above background levels was not detectable in cells that had been transfected with HSV-TK only, while no cytotoxic activity was conferred to cells expressing only Luc.

Cytotoxic and Luc activity in cells transiently transfected with the HSV-TK-Luc fusion construct were also compared to the respective activities in cells transiently transfected with equimolar amounts (50 ng – 2 μg DNA) of *HSV-TK *or *Luc *alone. The overall cytotoxic activity of the fusion construct proved to be 60% of that measured in cells transfected with *HSV-TK *alone. Representative curves for 2 μg and 0.1 μg of transfected DNA are shown in Figure 2A. Luc activity of the fusion protein was also lower compared with native Luc: light production in cells transfected with *HSV-TK-Luc *was 22% of that seen in cells transfected with *Luc *only (Figure 2B). A tight linear correlation of bioluminescence to cell kill was achieved with the fusion protein, suggesting that light emission can indeed be used as a measure for the cytotoxic effect of transgenic HSV-TK.

### Stable expression of the HSV-TK-Luc fusion gene in human glioma cells

Having demonstrated in transient transfection experiments that Luc could be employed as a reporter for monitoring the therapeutic effect of HSV-TK, U87MG cell clones stably expressing the HSV-TK-Luc fusion protein were generated by selection of transfected cells with geneticin. Comparison of 18 of these clones for Luc and cytotoxic activity revealed a good correlation between both enzymatic activities (R^2 ^= 0.79; p < 0.001, data not shown).

Enzymatic activity in the U87MG clone with both the highest Luc and HSV-TK activity was compared with U87MG clones expressing unfused HSV-TK or Luc. Cells stably expressing HSV-TK did not luminesce upon addition of D-Luciferin while Luc expressing cell clones were resistant to GCV mediated cell killing (data not shown). The dual function fusion protein compared favorably to the respective clones with the highest HSV-TK or Luc activity. Light production in the HSV-TK-Luc expressing cell clone was ~41% of that seen in Luc expressing U87MG cells while cytotoxic activity of the HSV-TK-Luc labeled U87MG clone was ~84% of that seen with the most active HSV-TK expressing U87MG clone (data not shown). Photon emission determined luminometrically was found to be linearly correlated with cell number over a range of at least 5 orders of magnitude (R^2 ^= 0.99; p < 0.001, data not shown). Photon emission from as few as 500 *intact *cells expressing the fusion construct was detectable by the CCD camera while the lower detection limit for the Luc expressing U87MG cell clone was 125 cells.

**Figure 3:**
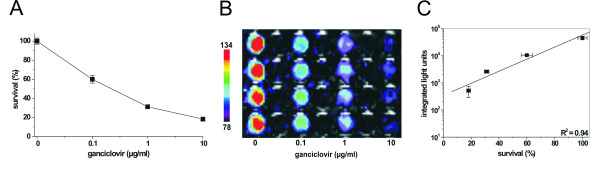
Correlation of light emission with cytotoxicity in intact U87MG glioma cells stably expressing the HSV-TK-Luc fusion construct and treated with GCV. (A) Cytotoxic activity as determined by MTT assay. (B) Bioluminescence imaging of quadruplicates of *intact *U87MG cells treated with the indicated amounts of GCV or left untreated (control). (C) Linear regression analysis of photon emission detected by the CCD camera plotted against cytotoxicity (R^2 ^= 0.94; p = 0.029).

We further examined whether the cytotoxic activity of the HSV-TK moiety could be visualized by monitoring light emission from *intact *cells that had been treated with GCV at different concentrations. Signals captured by the CCD camera showed a close correlation to the cytotoxic effect as measured by MTT assay (Figure 3, R^2 ^= 0.94; p = 0.029). These data demonstrate that both enzyme activities were also preserved in U87MG cells *stably *expressing the HSV-TK-Luc fusion construct.

### Correlation of HSV-TK with luciferase activity in vivo

The above high expresser U87MG cell clone was used for xenograft experiments in nude mice. For sensitivity testing, 2 × 10^3^, 2 × 10^4 ^and 2 × 10^5 ^cells were injected s.c. on the back of the animals. Although not palpable, 2 × 10^4 ^cells expressing the fusion construct were detected by the CCD camera immediately after injection (= day 0), either when injected alone or mixed with 1.8 × 10^5 ^(90%) non-luminescent parental U87MG cells prior to injection, while 2 × 10^3 ^cells injected s.c. were not seen (data not shown). This high level of detectability by BLI proves the usefulness of the HSV-TK-Luc construct as a highly sensitive reporter *in vivo.*

**Figure 4:**
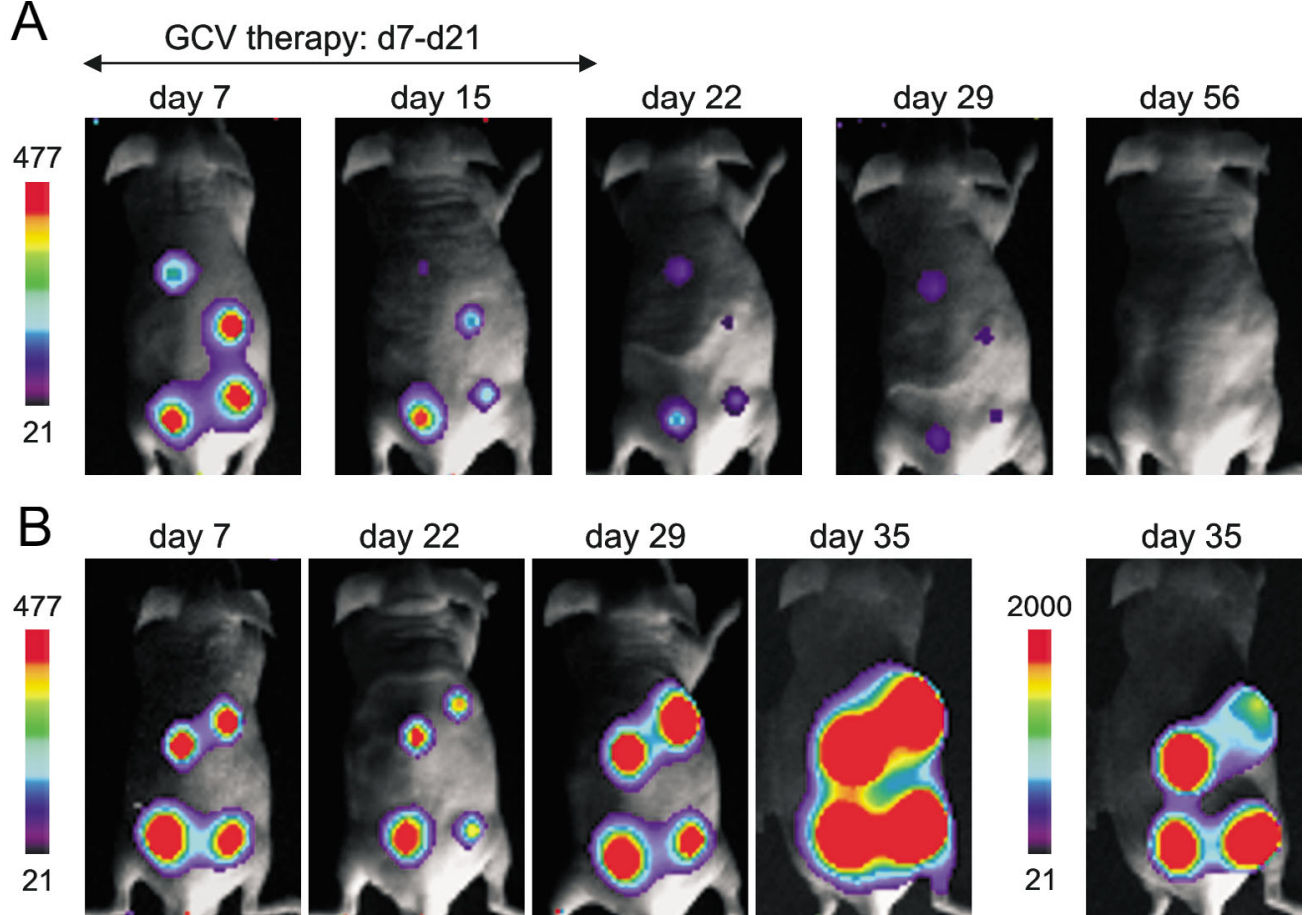
Bioluminescence imaging of nude mice carrying HSV-TK-Luc expressing U87MG gliomas. (A) Serial images from a mouse with 4 s.c. xenografts treated with GCV from day 7 to day 21 post tumor implantation and (B) saline treated control mouse, sacrificed on day 35 post tumor implantation due to massive tumor growth. The largest tumor in this mouse on the upper right back has already become necrotic. The day 35 image is also displayed at a broader grayscale range for better visualization of tumor localization. Note that control tumors also showed decreased light emission and a reduction in tumor size within the first 3 weeks post tumor implantation.

**Figure 5:**
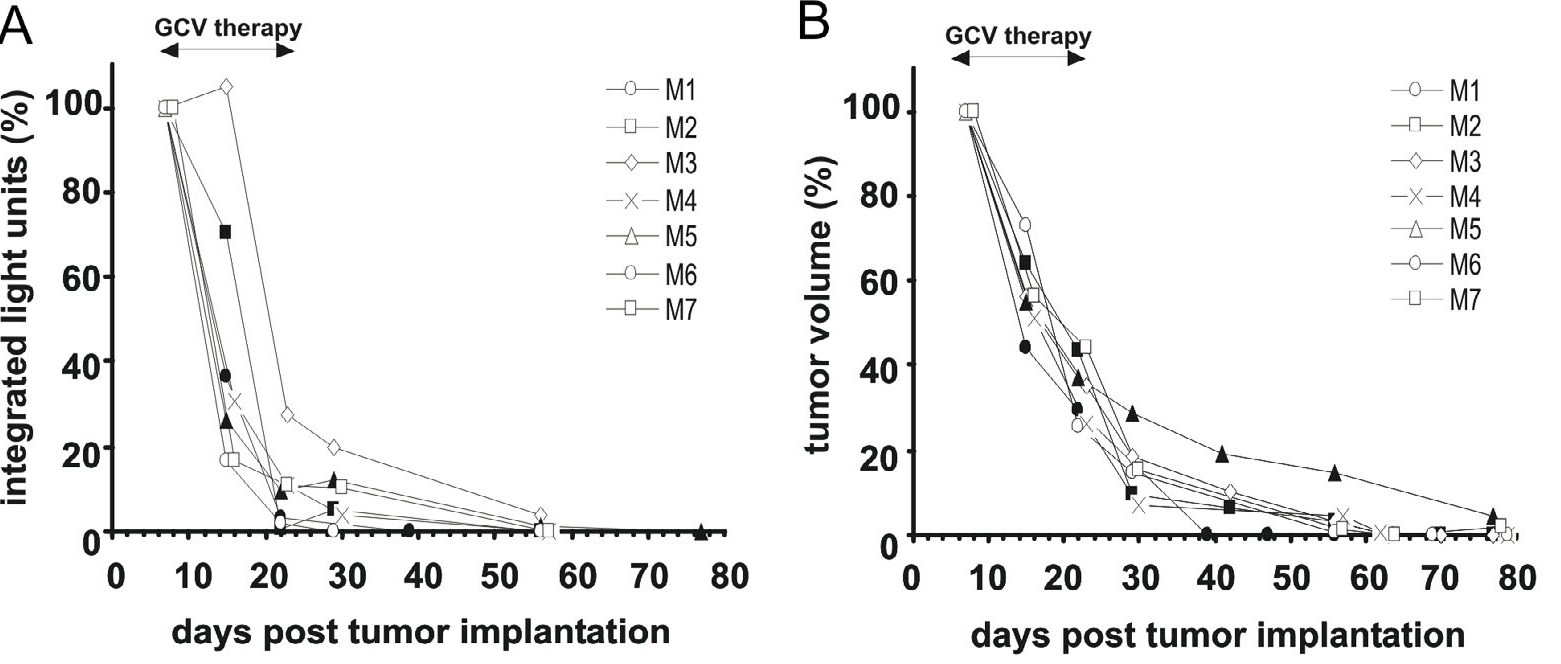
Comparison of (A) bioluminescence signals detected by the CCD camera and (B) tumor volumes in GCV-treated mice (n= 7, M1 – M7) harboring HSV-TK-Luc tagged U87MG glioma xenografts. GCV (60 mg/kg per day) was administered for 14 days starting at day 7 post tumor implantation. All mice were imaged at least on days 7, 15, 22, 29, and 56 post tumor implantation. BLI signals and tumor volumes at the beginning of therapy were set as 100%. Identical symbols in both graphs correspond to identical animals.

**Figure 6:**
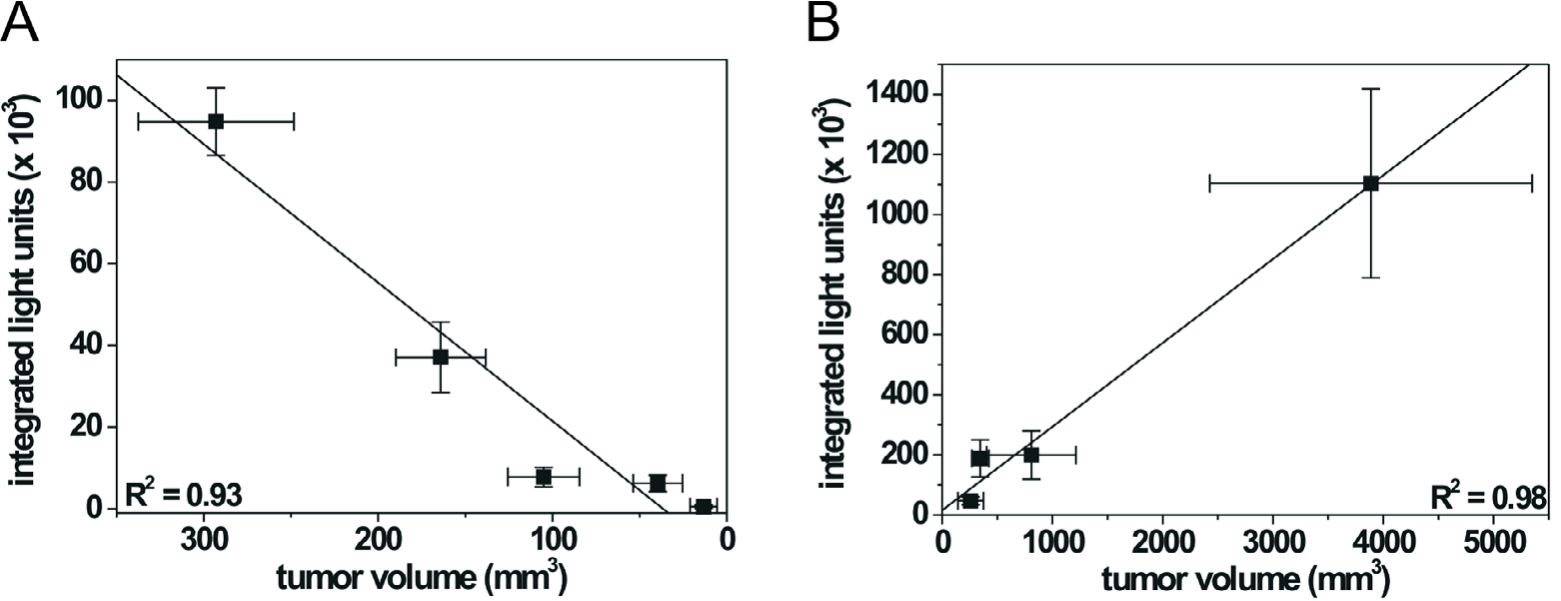
Linear regression analysis. Light emission as determined by the CCD camera was plotted against tumor volume. Mean values for all animals in a group along with the S.E.M are reported. (A) GCV treated mice: mice (n = 7) were imaged at start of therapy, after 1 and 2 weeks of GCV treatment, and 1 and 5 weeks after end of GCV therapy (R^2 ^= 0.93, p = 0.008). In some mice additional images were acquired (Figure 5), but these were not included in this plot. (B) Saline treated control mice: these mice (n = 3) were imaged on days 7, 22, 29, and 35 and were sacrificed after the last BLI due to massive tumor growth (R^2 ^= 0.98, p = 0.010).

For therapeutic studies, mice received injections with 2 × 10^6 ^HSV-TK-Luc labeled U87MG cells at four different sites on the back and the flanks, respectively (Figure 4). When tumors had reached a size of ~5 mm in diameter, a bioluminescence image was acquired and GCV therapy was initiated (n = 7). GCV treatment did not cause any significant toxicity and treated mice displayed normal patterns of food intake and physical activity. Control animals (n = 3) received saline injections. Initial tumor volumes in all mice were 309 ± 37 mm^3 ^and light intensity units (= counts) measured on day 7 were 122961 ± 22155. Serial measurements (during and after GCV therapy) of tumor volumes and integrated light intensity units within a region of interest (ROI) including all tumors were plotted against each other (Figure 5). Within the 2 weeks of GCV treatment, all 7 mice showed a rapid decline in photon emission from their tumors (mean decrease: 92 ± 7%, Figure 5A), which was accompanied by a somewhat slower decrease in tumor volume (65 ± 19%, Figure 5B). A linear regression analysis of mean tumor volumes in treated mice on days 7, 15, 22, 29, and 56 post tumor implantation plotted against the respective mean integrated light units is displayed in Figure 6A. Light emission and tumor volumes correlated closely with each other (R^2 ^= 0.93; p = 0.008), thus confirming our cell culture data (Figures 1 and 3).

Regarding therapeutic efficacy in these mice on an individual basis, photon emission and tumor volumes showed a significant correlation, with R^2 ^values ranging from 0.78 to 0.96 and p ranging from 0.004 to 0.047, except for one mouse (R^2 ^= 0.731; p= 0.065). In this mouse a significant correlation between light emission and tumor volume could be demonstrated, when tumor volumes were plotted against the *maximum *light emission within a ROI (R^2 ^= 0.81; p = 0.037) instead of integrated light units. In general, integrated light units within a ROI correlated closely with maximum light emission from this ROI: correlation coefficients (R^2^) for all tumors in treated mice varied between 0.97 and 0.99, all p values were <0.003.

Five weeks after end of GCV therapy (day 56) light emission was no longer detectable in 5 of the 7 GCV-treated mice, while in 4 of them small residuums at the tumor site were still visible. Two mice still showed very weak light emission from one of their flank tumors which also disappeared in subsequent imaging studies. All GCV treated mice survived and tumor recurrence was not observed until closure of the study at day 90 post tumor implantation.

The 3 untreated control mice were imaged on days 7, 22, 29, and 35 post tumor implantation and had to be sacrificed on weeks 5 to 6 post cell injection due to massive tumor growth. Although 2 tumors with relatively strong light emission on day 7 post tumor implantation regressed within 4 weeks in one of the control mice, overall tumor growth in all control animals plotted against light emission from these tumors still showed a tight correlation (R^2 ^= 0.98; p = 0.010; Figure 6B). Within 4 weeks, tumor volumes increased by ~11-fold while photon emission concomitantly rose ~6-fold. When tumors had become very large (>12–15 mm in diameter) further increase in light emission was much less than the increase in volume. This was mirrored by a less stringent linear correlation of BLI signal and tumor size in large tumors.

The control mouse shown in Figure 4B serves as an example: on day 35 the tumor on the right upper back shows an attenuated bioluminescent signal although being the largest tumor. While linear regression analysis demonstrated a very good correlation of tumor volume to light emission for the other 3 tumors (R^2 ^= 0.99 and p = 0.002 for tumors on the back and the right flank; R^2 ^= 0.98 and p = 0.011 for the tumor on the left flank), R^2 ^was 0.90 (p = 0.050) for this large tumor. When serially determined *maximum *light emission was used for quantification in this particular tumor, R^2 ^dropped to 0.37 (p = 0.4).

**Figure 7:**
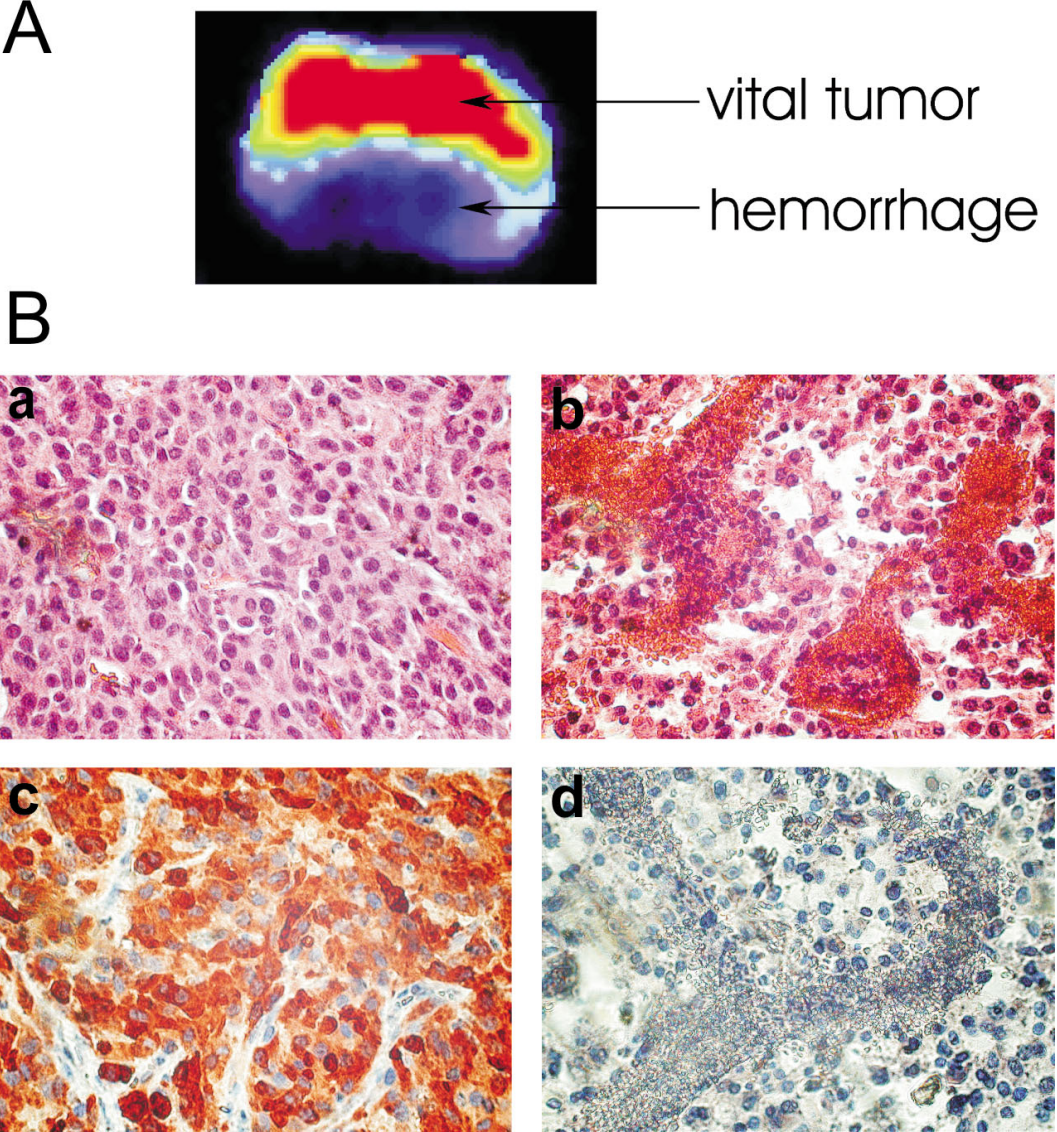
*Ex vivo *bioluminescence imaging and histological analysis of a large HSV-TK-Luc tagged U87MG glioma in a control mouse. (A) Bioluminescence image (exposure time 10 min) of the freshly explanted tumor after D-Luciferin injection in the mouse prior to sacrifice. The tumor was cut in the middle and placed with the cut side facing the CCD camera. One part of the tumor was obviously hemorrhagic while the other part looked vital. Photon emission displayed in pseudocolors precisely reflected the macroscopic findings. (B, a and b) HE staining of paraffin sections from the same tumor as seen in (A) vital tumor region (a); hemorrhagic and necrotic tumor area (b); (B, c and d) immunohistochemistry on corresponding sections using a polyclonal anti-Luc antibody; vital tumor area (c) and hemorrhagic and necrotic region (d) with only scarce positive staining. Original magnification × 400.

When control mice were sacrificed, tumors were explanted, and immediately reimaged. Bioluminescence imaging confirmed reduced light emission from hemorrhagic and necrotic areas in large tumors (Figure 7A). Immunohistochemical analysis of these tumor regions using a polyclonal anti-Luc antibody also showed a relatively scarce positive staining of necrotic areas as compared to areas with strong photon emission (Figure 7B).

## Discussion

This study demonstrates that firefly luciferase is a valuable tool for monitoring noninvasively the efficacy of the prodrug activating system *HSV-TK*/ GCV in cell culture and *in vivo*. The HSV-TK-Luc fusion protein was successfully used in a brain tumor animal model for serial and sensitive real time quantification of the cytotoxic effect of HSV-TK by BLI.

### Correlation of enzymatic activities

Fusion of two enzymes is the only way to guarantee stoichiometric, and thus correlated expression of both fusion partners. We chose this approach because coexpression of two separate transgenes from either one or separate promoters has been reported to result in severely impaired gene expression, e.g. due to inefficient internal ribosome entry site (IRES)-mediated translation [19] or to promoter interference [20].

The HSV-TK protein contains several nuclear targeting signals and is usually located predominantly in the nucleus [21]. The enzyme may form a homodimer, and it has been proposed that only dimeric HSV-TK is transported to the nucleus [21]. On the other hand, in the commercially available firefly Luc we have used, the peroxisomal targeting sequence present in wild type Luc has been removed to ensure strict cytosolic compartmentalization, and thus reliable reporter function [17]. Fusing both enzyme moieties together resulted in a protein with predominant localization in the cytosol, as shown by the immunohistochemical analysis of HSV-TK-Luc expressing glioma cells (Figure 7B). In contrast, we have shown previously that fusion of the 27 kDa protein EGFP to HSV-TK allowed for predominant nuclear transfer of the enzyme while resulting in only minor loss of cytotoxic activity [22], suggesting that cytosolic localization and/ or reduced homodimer formation of the HSV-TK-Luc fusion protein may have some impact on its cytotoxic activity.

A decrease in HSV-TK activity of up to 80% compared to unfused HSV-TK has also been observed by Ray et al. when fusing the enzyme to *Renilla *Luc [23]. N2A neuroblastoma cells harboring the fusion construct could be detected by PET and BLI in nude mice, but the *cytotoxic *activity of HSV-TK was not examined in the study. *Renilla *Luc activity in the above construct was found to be ~6 – 8-fold higher than seen with its unfused counterpart. As this enzyme is structurally unrelated to firefly Luc and has a lower molecular weight (36 kDa vs. 62 kDa), the study cannot be directly compared to our data. Notably, the authors mention that their attempt to fuse HSV-TK to firefly Luc resulted in a "poorly active" fusion protein [23].

Recently, the generation of several triple fusion proteins for imaging with different modalities was reported by two groups [24,25]. These triple reporters consisted of wild type or mutated HSV-TK, a fluorescent protein (EGFP, DsRed2, or monomeric red fluorescent protein (mRFP)) and firefly Luc [24,25] or *Renilla *Luc [25], respectively. Both groups showed that such triple fusion constructs could be used for simultaneous imaging *in vivo *with bioluminescence, fluorescence, and PET. Cytotoxicity generated by enzymatic conversion of prodrug by HSV-TK was however not measured in either of the above studies.

Ponomarev et al. [24] did not present data on the *correlated *expression of the three reporters within the triple fusion protein (HSV-TK-EGFP-Luc), nor were the activity levels of the different fusion partners compared to those of their unfused counterparts. Despite these limitations, the study confirms that Luc remains functional if fused N-terminally to other proteins, and that enzymatic activity is sufficient for *in vivo *BLI.

Ray et al. [25] compared the enzymatic and fluorescent activities of several triple fusion constructs transiently transfected into 293 cells to the respective activities of the unfused proteins. The described fusion proteins contained either *Renillla *or firefly Luc at the NH_2_-terminus, followed by a fluorescent protein and a mutated HSV-TK enzyme. This orientation of the fusion partners as well as the use of mutated HSV-TK optimized for use with PET limits the direct comparison of the presented data to our results. Bioluminescent activity of the 4 fusion constructs containing firefly Luc was reduced to 22 – 63% of the activity of unfused Luc, which is similar to our findings when expressing HSV-TK-Luc in U87MG glioma cells. One of the 4 constructs (Luc-mRFP-mutant HSV-TK) fully retained HSV-TK PET reporter activity while in the others HSV-TK activity (as assessed by intracellular radiotracer accumulation) was reduced to 30 – 61% of the activity of the corresponding unfused enzyme. This is in line with our findings when expressing HSV-TK-Luc transiently in U87MG glioma cells.

Although it seems attractive to perform BLI with different luciferase enzymes, the following facts argue in favor of firefly Luc instead of *Renilla *Luc: (1) light emission of *Renilla *Luc peaks at 480 nm and thus shows only limited tissue penetration, (2) coelenterazine, the *Renilla *Luc substrate is prone to autoluminescence, resulting in high background if injected i.p. [26], (3) coelenterazine transport (and thus the bioluminescent signal) is modulated by the multidrug resistance MDR1 P-glycoprotein, a protein known to be overexpressed in cancer cells [27], and (4) coelenterazine is much more expensive than D-Luciferin.

Iyer et al. [28] examined noninvasive imaging using PET and BLI in CD-1 mice after simultaneous i.v. delivery of the *HSV-TK *and *Luc *genes residing on different plasmids. The time point of peak activity of both reporters differed by ~19 hours, most likely due to differences in half lives of the two enzymes. This finding supports our approach of expressing both enzymes as one molecule as this should greatly diminish differences in protein stability.

Attenuation of both enzymatic activities is most likely a result of steric hindrance and might be substantially reduced by selecting another linker sequence. Longer intervening sequences as well as introduction of flexible polyglycine linkers may contribute to an increase in enzyme activity [23,29].

Recently, De et al. [15] introduced into a lentiviral vector a mutant HSV-TK (optimized for PET imaging) and firefly Luc, separated by an IRES sequence. N2a neuroblastoma cells stably transduced with this construct and implanted s.c. into nude mice showed correlated expression of both enzymes as verified by PET and BLI (R^2 ^= 0.86). In contrast to our study, GCV treatment *in vivo *resulted in a decrease in light emission while the tumors continuously grew in size. Most likely, this reflects the relatively poor cytotoxic activity of the mutant HSV-TK used in these experiments, implying that engineered *HSV-TK *optimized for use as a PET reporter may not retain its full cytotoxic activity when substrates such as GCV are used. Data on the cytotoxic potential of the virus construct in cell culture were not presented.

### Cytotoxic effects of the fusion construct

We show here for the first time that a HSV-TK-Luc fusion protein in conjunction with GCV treatment can confer a curative effect on glioma bearing animals. While HSV-TK-Luc expressing glioma cells in culture were not killed completely when using GCV concentrations of up to 10 μg/ml, xenografts consisting of these cells were fully eliminated in all GCV treated mice. It has been shown by several groups that HSV-TK expressing tumor cells can elicit an antitumor immune reaction even in immunocompromised animals such as nude mice, most likely mediated by natural killer (NK) cells, activated in vivo by GCV induced cell killing [30,31]. We suggest that such an immune response may also have contributed to the elimination of HSV-TK-Luc expressing U87MG glioma cells in GCV-treated mice. This issue could be further addressed by in vivo depletion of NK cells through administration of appropriate antibodies [30].

### Optical detection of transgene expression

Our study used a subcutaneous glioma model for "proof of concept" to allow for simultaneous bioluminescence imaging and measurement of tumour size by caliper. HSV-TK-Luc expressing U87MG glioma cells were also detected by the CCD camera after inoculation of 2 × 10^6 ^cells intracerebrally in nude mice (data not shown), confirming the high sensitivity of BLI. Indeed, it has already been demonstrated in a murine orthotopic pituitary tumor model that bioluminescent light can travel through skull [32].

The cooled CCD camera system we used allows for quantification of emitted light. Some authors suggested that the level of transgene expression could be more reliably quantified by maximum light emission than by integrated light units within a ROI [33]. Although quantification is important if strategies for transgene delivery are to be examined, a systematic comparison of these two parameters in BLI has not been published until now. This prompted us to analyze these parameters in more detail in our study.

If maximum and integrated light units within a ROI over single tumors were compared, we consistently found that both parameters tightly correlated with tumor size in tumors up to ~1 cm in diameter. In larger tumors (maximum diameter examined = 2 cm) the increase in size was in general less closely correlated with both integrated and maximum light units, but light emission from a ROI drawn over the entire tumor was still far more accurately mirroring tumor growth than maximum light units emitted from this region. With the increase in tumor thickness, maximum light emission from the tumor core is reduced due to necrosis, light scattering, and reduced supply of oxygen and D-Luciferin to tumor cells. On the other hand, the increase in tumor length and width is better reflected by light signals integrated over the entire tumor area, while a concomitant change in maximum light signal does not necessarily have to occur. Therefore, *integrated *light signals emitted from tumor ROIs seem to be the measure of choice for serial imaging of transgene expression in growing tumors. The fact that tumor volume and photon emission are less tightly correlated in large tumors as compared to smaller ones implies that photon emission reflects mainly the presence of *viable *tumor cells within a tumor and is a more precise measure for cytotoxic efficacy than tumor size, as has also been suggested by others [34,35].

Herpes simplex virus type I thymidine kinase has also been used as a reporter gene for monitoring therapeutic success with PET [11,36]. However, using optical imaging methods to quantify transgene expression has several advantages. The much greater sensitivity of BLI as compared to PET (10^-15 ^– 10^-17 ^vs. 10^-11 ^– 10^-12 ^mole/L of reporter probe are detectable, [13]) in combination with very low background signals render this imaging method particularly attractive for studying therapeutic strategies in animal models of cancer. In addition, high costs, the need for a radiopharmacy and considerable technical experience currently preclude widespread application of PET in experimental gene therapy of cancer.

## Conclusions

We showed that therapeutic efficacy of a suicide gene/ prodrug activating system can be accurately monitored *in vivo *by BLI, when the bioluminescent reporter luciferase is fused in frame to the therapeutic gene *HSV-TK*. We used a clonal human glioma cell line stably expressing the HSV-TK-Luc fusion construct, thus guaranteeing high level transgene expression. Despite the somewhat attenuated activity of both fusion partners, a high degree of cytotoxicity by HSV-TK mediated GCV bioactivation as well as strong bioluminescent signals upon administration of D-luciferin were consistently demonstrated. In order to mirror more closely *in vivo *gene therapy of malignant brain tumors, experiments are underway to insert the *HSV-TK-Luc *fusion gene (and an improved version of it) into appropriate viral vectors and subsequently use them for treatment of orthotopically established gliomas in mice. Serial assessment of transduction levels, transgene localization and time course of fusion gene expression in living animals by BLI can aid in developing more potent gene therapy vectors for treatment of malignant glioma.

## List of abbreviations

CCD, charged coupled device; CMV, cytomegalovirus; cps, counts per second; EGFP, enhanced green fluorescent protein; GCV, ganciclovir; HSV-TK, herpes simplex virus type 1 thymidine kinase; Luc, luciferase; mRFP, monomeric red fluorescent protein, PET, positron emission tomography; ROI, region of interest; SD, standard deviation, SEM, standard error of the mean.

## Competing interests

None declared.

## Authors contributions

AS constructed the plasmids, performed the animal experiments together with CT and set up the in vitro assays. CT and SJ participated in the cell culture experiments and enzymatic assays. SJ and AS carried out the immunohistochemical studies. NGR and AS designed the experiments and evaluated the data. All authors have read and approved the manuscript.

## References

[B1] Ram Z, Culver KW, Walbridge S, Blaese RM, Oldfield EH (1993). In situ retroviral-mediated gene transfer for the treatment of brain tumors in rats. Cancer Res.

[B2] Izquierdo M, Cortes M, de Felipe P, Martin V, Diez-Guerra J, Talavera A, Perez-Higueras (1995). Long-term rat survival after malignant brain tumor regression by retroviral gene therapy. Gene Ther.

[B3] Mizuno M, Yoshida J, Colosi P, Kurtzman G (1998). Adeno-associated virus vector containing the herpes simplex virus thymidine kinase gene causes complete regression of intracerebrally implanted human gliomas in mice, in conjunction with ganciclovir administration. Jpn J Cancer Res.

[B4] Ram Z, Culver KW, Oshiro EM, Viola JJ, DeVroom HL, Otto E, Long Z, Chiang Y, McGarrity GJ, Muul LM, Katz D, Blaese RM, Oldfield EH (1997). Therapy of malignant brain tumors by intratumoral implantation of retroviral vector-producing cells. Nat Med.

[B5] Shand N, Weber F, Mariani L, Bernstein M, Gianella-Borradori A, Long Z, Sorensen AG, Barbier N (1999). A phase 1 – 2 clinical trial of gene therapy for recurrent glioblastoma multiforme by tumor transduction with the herpes simplex thymidine kinase gene followed by ganciclovir. GLI 328 European-Canadian study group. Hum Gene Ther.

[B6] Rainov NG (2000). A phase III clinical evaluation of herpes simplex virus type 1 thymidine kinase and ganciclovir gene therapy as an adjuvant to surgical resection and radiation in adults with previously untreated glioblastoma multiforme. Hum Gene Ther.

[B7] Harsh GR, Deisboeck TS, Louis DN, Hilton J, Colvin M, Silver JS, Qureshi NH, Kracher J, Finkelstein D, Chiocca EA, Hochberg FH (2000). Thymidine kinase activation of ganciclovir in recurrent malignant gliomas: a gene-marking and neuropathological study. J Neurosurg.

[B8] Sandmair AM, Loimas S, Puranen P, Immonen A, Kossila M, Puranen M, Hurskainen H, Tyynela K, Turunen M, Vanninen R, Lehtolainen P, Paljarvi L, Johansson R, Vapalahti M, Yla-Herttuala S (2000). Thymidine kinase gene therapy for human malignant glioma, using replication-deficient retroviruses or adenoviruses. Hum Gene Ther.

[B9] Rainov NG, Kramm CM, Aboody-Guterman K, Chase M, Ueki K, Louis DN, Harsh GR, Chiocca A, Breakefield XO (1996). Retrovirus-mediated gene therapy of experimental brain neoplasms using the herpes simplex virus-thymidine kinase/ ganciclovir paradigm. Cancer Gene Ther.

[B10] Kruse CA, Roper MD, Kleinschmidt-DeMasters BK, Banuelos SJ, Smiley WR, Robbins JM, Burrows FJ (1997). Purified herpes simplex virus thymidine kinase retrovector™ particles. I. In vitro characterization, in situ transduction efficiency, and histopathological analyses of gene therapy-treated brain tumors. Cancer Gene Ther.

[B11] Gambhir SS (2002). Molecular imaging of cancer with positron emission tomography. Nat Rev Cancer.

[B12] Ross BD, Chenevert TL, Rehemtulla A (2002). Magnetic resonance imaging in cancer research. Eur J Cancer.

[B13] Massoud TF, Gambhir SS (2003). Molecular imaging in living subjects: seeing fundamental biological processes in a new light. Genes Dev.

[B14] Wetterwald A, Van der Pluijm G, Que I, Sijmons B, Buijs J, Karperien M, Löwik CW, Gautschi E, Thalmann GN, Cecchini AG (2002). Optical imaging of cancer metastasis to bone marrow. Am J Pathol.

[B15] De A, Lewis XZ, Gambhir SS (2003). Noninvasive imaging of lentiviral-mediated reporter gene expression in living mice. Mol Ther.

[B16] Lyons RM, Forry-Schaudies S, Otto E, Wey C, Patil-Koota V, Kaloss M, McGarrity GJ, Chiang YL (1995). An improved retroviral vector encoding the herpes simplex virus thymidine kinase gene increases antitumor efficacy in vivo. Cancer Gene Ther.

[B17] Groskreutz DJ, Sherf BA, Wood KV, Schenborn ET (1995). Increased expression and convenience with the new pGL3 luciferase reporter vectors. Promega Notes Mag.

[B18] Lee SY, Wang Z, Lin CK, Contag CH, Olds LC, Cooper AD, Sibley E (2002). Regulation of intestine-specific spatiotemporal expression by the rat lactase promoter. J Biol Chem.

[B19] Mizuguchi H, Xu Z, Ishii-Watabe A, Uchida E, Hayakawa T (2000). IRES-dependent second gene expression is significantly lower than cap-dependent first gene expression in a bicistronic vector. Mol Ther.

[B20] Ginn SL, Fleming J, Rowe PB, Alexander IE (2003). Promoter interference mediated by the U3 region in early-generation HIV-1-derived lentivirus vectors can influence detection of transgene expression in a cell-type and species-specific manner. Hum Gene Ther.

[B21] Degreve B, Esnouf R, De Clercq E, Balzarini J (1999). Characterization of multiple nuclear localization signals in herpes simplex virus type 1 thymidine kinase. Biochem Biophys Res Commun.

[B22] Söling A, Simm A, Rainov NG (2002). Intracellular localization of Herpes simplex virus type 1 thymidine kinase fused to different fluorescent proteins depends on choice of fluorescent tag. FEBS Lett.

[B23] Ray P, Wu AM, Gambhir SS (2003). Optical bioluminescence and positron emission tomography imaging of a novel fusion reporter gene in tumor xenografts of living mice. Cancer Res.

[B24] Ponomarev V, Doubrovin M, Serganova I, Vider J, Shavrin A, Beresten T, Ivanova A, Ageyeva L, Tourkova V, Balatoni J, Bornmann W, Blasberg R, Tjuvajev G (2004). A novel triple-modality reporter gene for whole-body fluorescent, bioluminescent, and nuclear noninvasive imaging. Eur J Nucl Med Mol Imaging.

[B25] Ray P, De A, Min JJ, Tsien RY, Gambhir SS (2004). Imaging tri-fusion multimodality reporter gene expression in living subjects. Cancer Res.

[B26] Wang W, El-Deiry WS (2003). Bioluminescent molecular imaging of endogenous and exogenous p53-mediated transcription in vitro and in vivo using an HCT116 human colon carcinoma xenograft model. Cancer Biol Ther.

[B27] Pichler A, Prior JL, Piwnica-Worms D (2004). Imaging reversal of multidrug resistance in living mice with bioluminescence: MDR1 P-glycoprotein transports coelenterazine. Proc Natl Acad Sci.

[B28] Iyer M, Berenji M, Templeton NS, Gambhir SS (2002). Noninvasive imaging of cationic lipid-mediated delivery of optical and PET reporter genes in living mice. Mol Ther.

[B29] Rogulski KR, Kim JH, Kim SH, Freytag SO (1997). Glioma cells transduced with an Escherichia coli CD/HSV-1 TK fusion gene exhibit enhanced metabolic suicide and radiosensitivity. Hum Gene Ther.

[B30] Hall SJ, Sanford MA, Atkinson G, Chen SH (1998). Induction of potent antitumor natural killer cell activity by herpes simplex virus-thymidine kinase and ganciclovir therapy in an orthotopic mouse model of prostate cancer. Cancer Res.

[B31] Bi W, Kim YG, Feliciano ES, Pavelic L, Wilson KM, Pavelic ZP, Stambrook PJ (1997). An HSVtk-mediated local and distant antitumor bystander effect in tumors of head and neck origin in athymic mice. Cancer Gene Ther.

[B32] Vooijs M, Jonkers J, Lyons S, Berns A (2002). Noninvasive imaging of spontaneous retinoblastoma pathway-dependent tumors in mice. Cancer Res.

[B33] Wu JC, Sundaresan G, Iyer M, Gambhir SS (2001). Noninvasive optical imaging of firefly luciferase reporter gene expression in skeletal muscles of living mice. Mol Ther.

[B34] Nyati MK, Symon Z, Kievit E, Dornfeld KJ, Rynkiewicz SD, Ross BD, Rehemtulla A, Lawrence TS (2002). The potential of 5-fluorocytosine/cytosine deaminase enzyme prodrug gene therapy in an intrahepatic colon cancer model. Gene Ther.

[B35] Mendel DB, Laird AD, Xin X, Louie SG, Christensen JG, Li G, Schreck RE, Abrams TJ, Ngai TJ, Lee LB, Murray LJ, Carver J, Chan E, Moss KG, Haznedar JO, Sukbuntherng J, Blake RA, Sun L, Tang C, Miller T, Shirazian S, McMahon G, Cherrington JM (2003). In vivo antitumor activity of SU11248, a novel tyrosine kinase inhibitor targeting vascular endothelial growth factor and platelet-derived growth factor receptors: determination of a pharmacokinetic/ pharmacodynamic relationship. Clin Cancer Res.

[B36] Jacobs A, Voges J, Reszka R, Lercher M, Gossmann A, Kracht L, Kaestle C, Wagner R, Wienhard K, Heiss WD (2001). Positron-emission tomography of vector-mediated gene expression in gene therapy for gliomas. Lancet.

